# Exploring the acceptability and feasibility of a whole school approach to physical activity in UK primary schools: a qualitative approach

**DOI:** 10.1186/s12889-022-14647-y

**Published:** 2022-11-30

**Authors:** Gareth Jones, Kim Longbon, Sarah Williams

**Affiliations:** 1 Academy of Sport and Physical Activity, Health and Wellbeing Department, Sheffield Hallam University, Sheffield, UK; 2 Department of Teacher Education, College of Social Sciences and Arts, Sheffield Hallam University, Sheffield, UK

**Keywords:** Physical activity, Whole school approach, Health, System change, Qualitative methods

## Abstract

**Background:**

UK Children generally fail to meet physical activity (PA) recommendations. Whole school approaches (WSA) have the potential to impact large numbers of children due to their ubiquitous nature for school wide implementation, however there is limited knowledge regarding primary school PA WSA implementation in the UK. This study aimed to investigate the acceptability and feasibility of a PA WSA in the UK.

**Methods:**

Semi structured interviews explored research aims with participants. A qualitative description approach was adopted and data were analysed using thematic analysis to draw codes and themes from the data.

**Results:**

Thirteen primary school senior leadership team (SLT) and Physical Education (PE) leads were interviewed. A PA WSA was found acceptable by all participants. Implementation, however, was questioned when other significant mechanisms were not in place. A PA WSA aided prioritisation and planning of PA provision, providing a holistic overview of all key areas of PE, school-sport and PA (PESSPA). Due to the high acceptability but dependent feasibility of a PA WSA, it is recommended that PA WSAs align with whole-school health policy and improvement plans to advance implementation. Future research, however, is needed to explore how this method is best implemented as additional interventions may also be required to promote the prioritisation of the PA agenda due to the importance of SLT backing for implementation being paramount, as results highlight.

**Conclusions:**

PA WSAs aid awareness, understanding and planning of school wide PESSPA provision, however their implementation in complex. Having SLT support and an appropriately resourced PE lead maximised the impact and utility of a PA WSA.

**Supplementary Information:**

The online version contains supplementary material available at 10.1186/s12889-022-14647-y.

## Introduction

UK chief medical officer (CMO) recommends children achieve 60 min of daily moderate-to-vigorous physical activity (PA), with at least 30 min delivered at school [1]. PA aids the prevention of childhood obesity [2], chronic disease [3], improved physical, mental, and social health [4–6], and academic function and performance [7–9]. Worryingly, less than half of UK children achieve CMO guidelines [10], following an international trend [11, 12].

School PA provision significantly impacts children’s PA due to the length of the school day [13]. School based PA also supports large and diverse populations of children [14, 15], aiding wide reaching lifelong PA behaviour adoption [16]. School PA interventions include classroom movement breaks, physically active learning, active travel, break time resources, and after school clubs [17, 18]. School staff however, struggle to implement sufficient PA interventions, citing barriers such as insufficient time, low confidence, weather restrictions, concerns of impact on learning time, and competing priorities [19–22]. Senior leadership team (SLT, e.g., head & deputy head teacher) and teachers understand the benefits of PA at school, often citing observed improvements in pupil concentration as a result of this, however, describe being overwhelmed by the barriers to implementation [19, 21].

The World Health Organisation recommend schools adopt PA policy to help aid the implementation of school PA initiatives [23]. Although there is good evidence for the impact school policy has on pupil PA behaviour [24], factors such as perceived policy priority, organisational leadership and coordinator qualities, understanding the school/pupil context, timetable constraints, intervention scope, resources (e.g., time, staff, equipment), social support (e.g., school governors), and teacher implementation ability, all impact PA policy adherence and maintenance [20, 25]. Thus, factors beyond PA interventions and policies are crucial for child PA behaviour. Public Health England therefore recommended schools implement whole school approach (WSA) programmes to aid children’s PA guideline adherence [26].

PA WSAs encourage multiple layers of a school network to work in partnership, including SLT, teachers, governors, and parents. This collaboration enables PA initiatives to have a meaningful impact on pupil health and academic performance [27, 28]. Research support that schools with a high PA WSA index score, have a greater frequency of children achieving recommended PA when compared to schools with a low PA WSA index score [11]. A potential benefit of WSA implementation resides in highlighting various opportunities to develop PA initiatives, a frequent barrier to intervention implementation reported by headteachers [21]. Limitations to WSAs however, include lack of time, resources, knowledge and social support, competing priority with academic focus’, and varying teacher intentions for PA promotion [15, 20, 29]. Insufficient communication between stakeholders (e.g., SLT, teachers, parents, pupils) can also cause friction for intervention implementation as priorities may not be congruent between groups [25]. Overall, WSAs demonstrate good utility to achieve increased PA adherence, especially if they are well communicated and utilised across the school [11, 25].

To date, there is limited research on PA WSAs in the UK, with research mainly being conducted in Northern America [20], or Europe [21, 29]. Thus, it is important to understand the acceptability and feasibility of a PA WSA within the UK. In addition, little is known about the key mechanisms that facilitate or inhibit the implementation of PA WSA. Using an example (Sheffield’s PESSPA Toolkit [29]), the current study aimed to explore the:

- Acceptability and feasibility of a PA WSA within the UK
- Key mechanisms that interact with the implementation of a PA WSA

## Methods

### Instrumentation

**Fig. 1:**
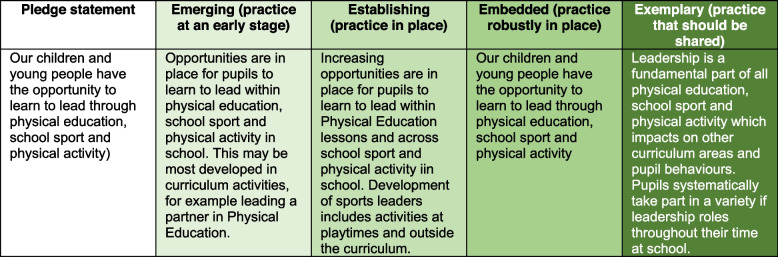
Example of one audit tool pledge statement and accompanying 4-point scale

The PESSPA Toolkit [30] is a PA WSA, providing an overview of primary school physical education, school sport and PA (PESSPA). The PESSPA Toolkit includes three key sections: 1) PESSPA rationale and evidence, 2) 14 pledge statements and guidance tool, and 3) an audit tool. The toolkit provides supplementary information of local organisations and resources. The 14 pledge statements are subdivided into each area of PESSPA (PESSPA *n* = 5, PE *n* = 4, SS *n* = 2, PA *n* = 3). The self-audit tool asks schools to rate themselves on a 4-point scale (Emerging, Establishing, Embedded, Exemplary). Each scale point of each pledge statement was accompanied with a detailed description (Fig. 1). The PESSPA toolkit was co-designed and developed by primary school sector stakeholders, including Learn Sheffield, School Sport Partnership (SSP) network, Sheffield Hallam University, and local primary PE leads and SLTs in an iterative design process. The group of stakeholders met once per half-term over an 18 month period to conceptualise, design, modify and agree on the content and presentation of the PESSPA Toolkit.

### Participants

Convenience sampling was utilised due to the time demand on participants during data collection, and being restricted to schools who showed willing to utilise the toolkit from the region the toolkit was developed (South Yorkshire, England). Recruitment took place via the regions SSP network, inviting primary schools to participate in the study, referencing the newly created PESSPA Toolkit. Fifty-two schools replied to the expression of interest. The research team provided detailed information regarding the project and the study requirements to interested schools. Ten schools responded and were approached for informed consent to participate. Any school who did not provide informed consent was excluded from the study. One school was lost during this process. The lead author liaised with the remaining schools PE leads and SLT’s, who agreed to review and utilise the PESSPA Toolkit as they saw fit in their school for the research study. Participants were then invited to interview.

Informed consent was gained from each participant, prior to data collection. Final analysis included nine primary schools from five of seven localities within the region, including a range of socio-economic status’. The lead author (GJ) conducted interviews with ten primary PE leads (female *n* = 5 (50%)) and three SLTs (head teacher *n* = 2, head of Key Stage 2 *n* = 1, female *n* = 3 (100%)).

### Procedure

A qualitative approach was adopted to address the research aims. Data can be used to inform new theory and practice within UK primary schools, highlighting the influencing factors that contribute to the implementation of a PA WSA. In order to assure quality of reporting, the consolidated criteria for reporting qualitative research (COREQ) 32-item checklist was used [31].

Semi-structured interviews, comprising open-ended questions based on previous literature and researcher knowledge, were conducted (Table 1) using a qualitative description approach. Interviews were either face-to-face or via online video call formats (e.g., Microsoft Teams), audio recorded, and transcribed verbatim. In total, 11 interviews were conducted with 13 participants. Nine interviews were one-to-one, and two interviews included two staff members simultaneously within the same school. Data collection took place between February – April 2020. The lead researcher (GJ) has sufficient knowledge and experience of collecting and analysing qualitative data. Interviews lasted an average of 42.51 min (SD = 13.4).

**Table 1:** Topic Guide example questions

Topic	Question
Physical Activity	1) Describe what physical activity means to your school.
PE	1) Please tell me about the meaning of PE to your school.
	a) How often do pupils participate in PE at your school?
	b) Is it the same across the school?
School Sport	1) Describe the opportunities there are for pupils to be involved in school sport at your school.
	a) Typology?
	b) Timing?
	c) Targeted programmes?
Staff Confidence, Opportunity and Motivation	1) Describe your thoughts on you and your staff’s confidence in being able to support pupil's activity at school.
	a)What enables / inhibits this confidence?
Whole School Approach, PESSPA Toolkit	1) Describe your initial thoughts on the PESSPA Toolkit.
	2) Describe your overall evaluation of the toolkit.
	3) Describe any impact, if any, you feel the PESSPA Toolkit will have on your schools provision of PESSPA.

### Data analysis

Data were analysed using both an inductive approach, based on participants’ personal accounts and experiences, and a deductive approach, based on the interview guide. Thematic analysis [32] was employed to analyse the transcripts via analysis software, NVivo V.11.4.3. All authors analysed a selection of transcripts using steps; (1) data familiarisation, (2) data coding and (3) generating themes, then met to discuss identified data codes and themes to create an analysis framework to add rigour and agreement of data interpretation. Lead author (GJ) continued to analyse the remaining transcripts based on this framework. Any additional themes/sub-themes were checked with the co-authors. The remaining thematic analysis steps were conducted, including (4) code and theme review, (5) define themes, and (6) report write up. Participants are identified by ID numbers and job role. Data were catalogued by job role to allow for exploration of the different participant views, perspectives and experiences.

## Results

All participants had a positive evaluation of PA, including PE leads and SLT members and perceived their school and pupils as ‘active’. Participants described the importance of a school in promoting active lifestyles, whilst highlighting variation in PESSPA provision throughout each school. Three global themes were identified, including eight sub themes (see Table 2).

**Table 2:** Global and Organising themes

Global theme	Organising themes
WSA impressions	A helpful tool
	Uncertainty of tool impact
PA WSA Facilitators	Leadership values
	Dedicated PE lead
	Supporting tools
PA WSA Barriers	Confidence
	Competing priorities
	Tradition of teaching and external structures

### Theme one: WSA impressions

#### A helpful tool

All participants, especially PE leads, reported a positive evaluation of the WSA document, suggesting it was a holistic tool for PESSPA provision. Participants emphasised how the toolkit aided awareness (*“it’s just going to make me more aware of the areas that I can improve”.* P1, PE lead) of the different areas of PESSPA, and prompted reflective practice (*“It just got us to take a step back and reflect.”,* P11, PE lead). This was often followed up by participants highlighting the tool’s utility to support action planning.

*“the toolkit itself… it saves you a job basically because by completing it you’re creating your own action plan.*” (P13, PE lead)

Almost all participants highlighted the PA WSA document supported a rationale for PESSPA provision and was a useful tool for reporting purposes (e.g., SLT, governors).

*“This would be a really, really good tool for the PE lead… for example if there was a lot of establishing and lower on it, go and take it to the head teacher and it’s a really good basis for them to say if Ofsted came in we would be struggling to show them that we are providing whole school sport and physical activity and we need to change this.”* (P11, PE lead).

#### Uncertainty of tool impact

A few participants highlighted uncertainty in the tool’s ability to create change (*“I don’t know what the impact will be”,* P4, PE lead), questioning if a PA WSA document was sufficient on its own to create change (*“if you’ve got the head and the senior leaders pushing it, without that, it’s not going to happen”*, P13, PE lead), with factors such as school culture and priorities, overshadowing implementation.

*“I do feel it is hopeful, but it depends on the school and the constraints of the school and how they’re choosing to use their resources and funds effectively.”* (P9, PE lead).

### Theme 2: PA WSA facilitators

#### Leadership values

All participants described PA culture as a key mechanism for PESSPA, linking the acceptance of a PA WSA to the school culture.

“*it’s the culture of the school and the ethos of the school, they [teachers] don’t shirk out of doing PE.”* (P9, PE lead).

All SLT members evaluated PESSPA positively and were supportive of the PESSPA Toolkit. Importantly, PE leads highlighted the significance of SLT values when discussing the PA culture. One participant referenced a change in leadership diverted focus from PESSPA, resulting in significantly reduced PESSPA provision. Three subsequent factors were highlighted when SLTs prioritised PESSPA; 1) improved PESSPA profile, 2) staff PESSPA provision (e.g., active classrooms), and 3) financial backing (e.g., PE specialist appointment).

*“We’ve always had from the very top, from the day I first started was a head who was very, very keen PE and kids being physical active… without that [SLT support] it’s not going to happen.”* (P13, PE lead).

Some participants discussed a school’s cultural context would influence leadership values (facilitate or inhibit), identifying contextual need and/or the cultural norms (e.g., parent or religious beliefs).

*“I think parental role is massive and that includes the financial side of it as well, a lot of my parents in my own school wouldn’t see themselves having the money to take the kids swimming or join a football club.”* (P13, PE lead).

#### Dedicated PE lead

All participants discussed the importance of PE lead’s role to implement SLT PESSPA values and utilise the PESSPA Toolkit. SLTs emphasised PE leads as the driving force behind a positive PA culture, needing to be knowledgeable (“[*PE lead*] *brought something a lot more deeper*”, P10, SLT) and passionate about PESSPA provision.

*“We’re very lucky in that we’ve got a very good, very competent PE leader and [they] are very good at coming to me... [They] keep very much on top of it, I feel quite confident that what needs to be happening is happening.”* (P3, SLT).

Almost all participants highlighted the PE lead’s role in the provision of staff PESSPA continued professional development (CPD) (“*like you need to give staff the option to have CPD…there needs to be … a PE coordinator who’s able to push on a regular basis and support.”* P13, PE lead), stating this positively impacted staff confidence and knowledge (*“We did our CPD for staff to kind of upskill them again and make them feel confident with teaching PE.”*, P11 PE lead). PE leads identified, sourced, and implemented required PESSPA CPD, as they had dedicated time and understanding of their school’s requirements.

*“My PE leader is incredibly skilled, very experienced. [They] work as a lead practitioner, and provided lots of training and is very supportive with staff.”* (P3, SLT).

One PE lead mentioned concerns of deskilling other staff; *“I do feel sometimes I’m deskilling the other staff by being there*.” (P7, PE lead). Overall however, participants highlighted the positive impact a PE lead has on other staff’s PESSPA skill, knowledge and confidence.

*“It’s also been about [PE lead] providing that high quality planning. So anything else that other teachers can pick that up and run with that. So they [staff] can enjoy teaching PE or be confident or take those risks or go out of their comfort zone.* (P10, SLT).

#### Supporting tools

PE leads highlighted two key factors to aid their role. Firstly, a variety of resources to cater for the variability of pupil and teacher preferences.

*“A lot of the staff use Go Noodle just as a five-minute brain break. We use Just Dance. So loads of different ideas… anything obviously that would suit their class.”* (P11, PE lead).

Secondly, participants discussed the benefit of external organisations (e.g., SSP’s and commercial organisations), stating it bolstered PESSPA provision and aided the implementation of a PA WSA.

*“We talk about all these opportunities for the children, if that organisation [local SSP] was to cease, this interview would be back to the bare minimum… without somebody driving sports leadership programmes, competitions, festivals, Change for Life, all these sort of things, they provide those opportunities.”* (P1, PE lead)*.*

### Theme three: PA WSA barriers

#### Confidence

The most frequently reported barrier was a perceived lack of staff confidence for PESSPA provision, reported to be routed in little knowledge and experience, a result of minimal PE teacher training.

*“Whatever you try to do to support them they feel a bit self-conscious themselves about doing PE. They don’t really feel like they’ve got the skills.”* (P3, SLT).

&

*“I think its experience as well. A lot of new staff in terms of their training, they’ve probably done one or two PE lessons being supported by a normal teacher, to then suddenly get 30 kids changed, outside, find the resources and have the desire… it’s an easy thing not to do.”* (P13, PE lead).

#### Competing priorities

All participants highlighted that although staff were perceived to evaluate PESSPA provision positively (“*they would say yes we want our children to be as active as possible*” P3, SLT), there was a wider cultural pressure for academic performance.

*“However much we try to prioritise PE, I think there’s so much pressure on reading, writing, maths… the time gets taken away from that opportunity to be physically active.”* (P3, SLT)*.*

This often resulted in PESSPA provision not being prioritised, especially around certain points of the academic year (e.g., SAT’s), *“I know from year 6 they very rarely do their second PE lesson until SATs is out of the way* (P7, PE lead).

#### Tradition of teaching and external structures

A few participants discussed how teachers have a ‘traditional model on teaching’, which was reported to negatively impact the provision of PESSPA, specifically active learning.

*“traditionally teachers saying, sit still, be quiet, listen to me, and actually them fidgeting is their indication that they need to move. So, it’s changing that shift in the mindset of children actually shouldn’t be still all of the time”* (P2, PE lead).

Some participants discussed how external structures (e.g., Ofsted), influenced PESSPA provision. Staff were alleged to modify PESSPA provision based on the perceived beliefs of these external structures.

*“I know the amount of pressure people are under and the pressure’s got much worse… you know, Ofsted, government, pressure on head teachers, offloading onto class teachers.”* (P8, PE lead)

## Discussion

### Main findings

The present study explored the acceptability and feasibility of a PA WSA, whilst simultaneously investigating key mechanisms to support implementation in primary schools. Acceptability of the PESSPA Toolkit were positive from all participants, highlighting its holistic nature for PESSPA provision and offered useful information and good practice. Benefits of this included action planning and a strengthened rationale for PESSPA initiatives. Uncertainty of the PA WSAs ability to create change, however, focused on the influence of competing priorities, staff confidence and intention, SLT values and school culture and context. Critically, PE leads appear important to help realise SLT values and implement actions derived from the PESSPA Toolkit. PE leads provided a pivotal role as a dedicated staff member to communicate between stakeholders (e.g., SLT, school staff) and offer wrap around support to other staff members (e.g., CPD/shadowing and resource provision). Regarding feasibility, PA WSA implementation hinged on SLT values, an adequate PE lead, and external support. For a school to adopt a PA WSA, it would seem multiple internal and external structures need to be in place that align in their value of PESSPA.

### Key interpretations

#### WSA acceptability and feasibility

The current study supports the acceptability, and to some degree, the feasibility of a PA WSA, adding further evidence of their potential benefit [11, 26]. Both SLT and PE leads evaluated the PESSPA Toolkit positively, paying specific interest to its holistic qualities, however some study bias needs to be considered here when interpreting the results, with most participants perceiving their school to have a positive PA culture. The PESSPA Toolkit enabled school SLT/PE leads to view all components of PESSPA provision and create strategies to improve their PESSPA offer. In line with previous literature, this is likely to support more children to live actively as strategic approaches aid PA provision [11]. Improved awareness of a schools PESSPA provision (strengths and weaknesses) enabled PE leads to create action plans for PESSPA provision, a known mechanism to improve provision [15].

The PESSPA Toolkit enabled some PE leads to align PESSPA provision with whole school improvement plans due to its holistic scope and was suggested that this would improve fidelity of PESSPA initiatives derived from the WSA. This integration between PESSPA provision and whole school improvement plans is a worthy solution to explore, as it could relieve previously suggested implementation and fidelity concerns [33] due to PA initiatives no-longer being an addition to what the school is already doing, rather how to achieve its overarching aims [21, 26, 27, 34, 35]. Current data suggests further investigation of how to achieve this however, would benefit. Lastly, the PESSPA Toolkit bolstered PE leads clarity, confidence, and justification for PESSPA action plans proposed to SLT, governors and external bodies (e.g., Ofsted). This further strengthened the implementation of PESSPA initiatives and the need for a PA WSA.

The PESSPA Toolkits feasibility was occasionally questioned due to perceived concerns to create change. On its own, the PA WSA was not seen significant enough to create change where other key PESSPA mechanisms were not in place (e.g., SLT backing). Various barriers to the utility of the PESSPA Toolkit were highlighted, including SLT prioritisation, classroom teacher confidence and intention, school context, and competing school priorities, supporting previous literature [15, 20]. Research highlights the importance of support from all school stakeholders, and without wide adoption, whole school initiatives are difficult to implement, calling into question the reliability of a WSA [29, 33], something the current study would support. There is little evidence to guide long term change for some recited barriers, such as SLT values and teacher confidence and intention. The adoption of a PA WSA may, therefore, be limited to schools where there is a positive PA culture and sufficient resources.

#### Mechanisms of change

The present study highlight key mechanisms to aid the implementation of a PA WSA. The PA culture, underpinned by SLT values had a significant impact on the utilisation of the PESSPA Toolkit due to their influence on the PESSPA profile and financial support. This impacted wider staff backing and resourcing for PESSPA provision in accordance with the SLT values. Understanding how to improve SLT prioritisation of PESSPA provision is a key future research topic, as evidence suggests that it is not necessarily that SLTs do not value PESSPA, rather, there are competing priorities contesting within limited capacity [21, 36]. Central government and Ofsted were highlighted as a significant influence on SLT priorities, thus potentially provides an opportunity to explore.

PE leads were seen with almost equal importance, providing an inhouse dedicated role (often specialist) with time and expertise to utilise the toolkit, helping realise SLT PESSPA values [33]. PE leads, although adopted by most schools, are not all equal, varying in experience and role parameters (e.g., dedicated or shared teaching role) with many PE leads not having specialist PE training [36]. Where the PE lead role is shared or not specialist trained, it was highlighted to lessen their impact for PA WSA adoption due to lacking appropriate resource (e.g., time, knowledge) to plan and implement initiatives. This supports previous literature highlighting the broad scope of a PE lead’s role [37]. Thus, the synergy between SLT PESSPA values and a proficient (dedicated/trained) PE lead was important for the utilisation of the PESSPA Toolkit, extending previous knowledge [11, 27, 35].

External organisations facilitated PESSPA provision, bolstering WSA implementation. This support aided PE leads acquisition of appropriate resources and provided opportunities to improve knowledge and experience as a PE lead. Given the importance of this mechanism, there is little evidence exploring their impact on school PESSPA provision.

Finally, school context was reported to influence the prioritisation of a PA WSA via influencing SLT values. Specifically, the deprivation level surrounding a school and the parental beliefs about PESSPA were highlighted [38]. Deprivation has previously been emphasised as a predictive factor in the provision of minutes of PE provided [39], emphasising that areas of low deprivation were associated with increased leisure time PA [40]. Due to the holistic nature of a PA WSA, it may therefore be especially valuable to demonstrate positive PESSPA values and utilise a PA WSA where a school is situated in an area of high deprivation as it is less likely that pupils are being sufficiently active.

### Study limitations

Some study limitations need to be considered when interpreting the findings. First, study results support some study bias, as all schools perceived their school as ‘active’. Second, study participants did not include all stakeholder opinions, such as staff teachers or governors. Third, it was a relatively small sample size, although five of the seven district localities were represented, and thus demonstrates a good geographical and sociodemographic spread. Finally, the generalisability of the results is limited to primary schools in the UK.

### Future research and implications

The current study demonstrates a positive response to a PA WSA from SLT and PE leads in the UK. In doing this, it highlights key future research topics. First, investigation of the acceptability and feasibility of a PA WSA in primary schools where the PA culture is not as positive or prioritised as in the current study, as this is still unknown. This could uncover additional barriers to implementation. Second, concentrate on exploring key mechanisms that support a school to utilise plans derived from a PA WSA. The present study highlights barriers for PA WSA plan implementation, thus gaining a more in-depth understanding of this would be of benefit. Third, to investigate mechanisms to increase SLTs prioritisation of PESSPA provision, as this is critical for PA WSA utilisation and there can be disparity between SLT attitudes (generally positive) and provision.

## Conclusion

Results support the acceptability, and to some degree, the feasibility for the implementation of a PA WSA in the UK, emphasising a favourable evaluation of the PESSPA Toolkit. Main benefits included increased awareness, scope and understanding of current PESSPA provision, improved ability to reflect and plan, whilst also providing a central rationale for PESSPA provision. Evidence suggests however, a PA WSA needs to be accompanied by SLT backing and a trained/passionate PE lead to create meaningful change. Additionally, understanding how to align a PA WSA with school improvement plans would benefit. Implementation of PA WSAs, therefore, is a complex strategy that requires further investigation and understanding to overcome relevant barriers to aid feasibility. Overall, SLT and PE leads believed a PA WSA was an acceptable strategy to support PESSPA provision in UK primary schools.

## Supplementary Information

**Additional file 1.** PESSPA staff interview schedule.

**Additional file 2.** PESSPA Toolkit 2022.

## Data Availability

The anonymised data generated and analysed during the current study are available via Sheffield Hallam Universities, SHURDA data deposit (https://shurda.shu.ac.uk/id/eprint/152).
