# Supplementary material for: Exploring the acceptability and feasibility of a whole school approach to physical activity in UK primary schools: a qualitative approach
Source: BMC Public Health. 2022 Nov 30;22:2236. doi: 10.1186/s12889-022-14647-y (PMC9713977; doi:10.1186/s12889-022-14647-y)
Supplement: Supplementary file 1 — Additional file 1. PESSPA staff interview schedule. [file 12889_2022_14647_MOESM1_ESM.docx]

**PESSPA First Interview - Staff**

Introduction:

General discussion about PE, School Sport and Physical Activity in your school. There are no right or wrong answers, we are just interested in exploring these themes.

Name: __________________________________ Age: ______________

Gender: ________________________ Job role: _________________________________

Part of the school SLT? Y / N

**Physical Activity overview**

Imagine the school day, where children were really active throughout the day, through active travel, active lessons, PE, active break times…

1. How would you feel about this?
2. What impact would this have on your school?
3. How do you feel this would influence the school?

PA

1. Describe what "physical activity" means to you?
2. Tell me about what you think of when PA is mentioned?
3. Describe what physical activity means to your school?
   - Do you think this ethos is generally reflected in practice? *How, why?*
4. What role does a school have in promoting physical activity to pupils?
   - How important is it that children are active during the school day?
   - How much opportunity do pupils have to be active whilst in school?
5. Thinking about break times for pupils;
   - Are pupils generally active?
     - How? Why / Why not?
   - Do you feel pupils have the appropriate resources to support activity?
   - Do the school playgrounds etc support PA?
   - Are there more opportunities your school could support with to help children’s activity?

PE

1. Describe what PE means to you?
2. Tell me about what you think of when PE is mentioned?
3. Please tell me about the meaning of PE to your school?
4. How often do pupils participate in PE at your school?
   - Is it the same across the school?
5. Describe the role, if any, the school has in promoting PE to pupils?
6. Are there any areas you feel your school could improve its PE offer to pupils?

SS

1. Describe what School Sport means to you?
2. Discuss what School Sport means, if any, to your school?
3. Describe the role schools have in promoting Sport to pupils, if any?
4. Describe the opportunities there are for pupils to be involved in school sport at your school?
   - Typology?
   - Timing?
   - Are there any targeted programmes?

**Staff**

Capability

1. Describe your thoughts on you and your staffs confidence in being able to support pupil's activity at school?
   - What enables / inhibits this confidence?
2. Discuss if you feel you have been supported by your school through CPD etc, to help activate pupils at your school?

Opportunity

1. Describe your thoughts on your schools provision of physical resources to help activate children at school? Staff time, pupil time & equipment etc.
   - equipment, playground/hall space, time, CPD training,
   - How and in which way?
   - Are there further opportunities?
2. Tell me about the social support / ethos in the school regarding PA, SS, PA
   - How does that shape your practice, if at all?

Motivation

1. Discuss the methods you use to activate children? Why these specifically?
2. How often would you say your children are active?
3. How motivated are you to activate pupils? How? Why?
4. How does your school ethos around activity, shape how you support children to be active at school?
5. What are the barriers to supporting activity at school?
6. What do colleagues and SLT think of activity at school (PE, SS, and general PA – active lessons, break times, before/after school clubs etc.

PESSPA

This section I would like to get your general thoughts on the PESSPA tool and the audit.

1. Describe your initial thoughts on the PESSPA toolkit?
   - What do you like about it / not like about it?
   - Useful / not useful – why?
   - When receiving it – what did you make of it?
2. Describe if anything has stood out to you about the toolkit?
3. When completing the audit, what thoughts did you have about the results?
   - Describe the process of completing it – useful? Time consuming? Etc.
4. Looking into the future, describe your thoughts on if the tool has been helpful or not?
   - What? How? Why, why not etc?
5. Describe if you feel the tool will, or will not, influence the school ethos around PE, SS and PA?
   - If so, how and in what way?
   - If not, why not?
6. What impact, if any, do you feel the PESSPA toolkit will have on your school in regard to PE, SS, PA?
   - How, why, in what way etc?
   - If not, why not?
7. Describe any changes, if any, that have been made in the short term from the PESSPA toolkit release?
   - How, why, in what way etc?
   - If not, why not?
8. Describe if you anticipate the PESSPA toolkit will help/hinder/make no difference to the amount of PA, or quality of PESSPA provision your pupils participate in at school?
   - If so, how, why?
   - If not, why not?

**Closing questions**

1. Is there anything else you would like to add?
2. Do you have any questions?
