# Supplementary material for: Exploring the acceptability and feasibility of a whole school approach to physical activity in UK primary schools: a qualitative approach
Source: BMC Public Health. 2022 Nov 30;22:2236. doi: 10.1186/s12889-022-14647-y (PMC9713977; doi:10.1186/s12889-022-14647-y)
Supplement: Supplementary file 2 — Additional file 2. PESSPA Toolkit 2022. [file 12889_2022_14647_MOESM2_ESM.pdf]

PHYSICAL EDUCATION

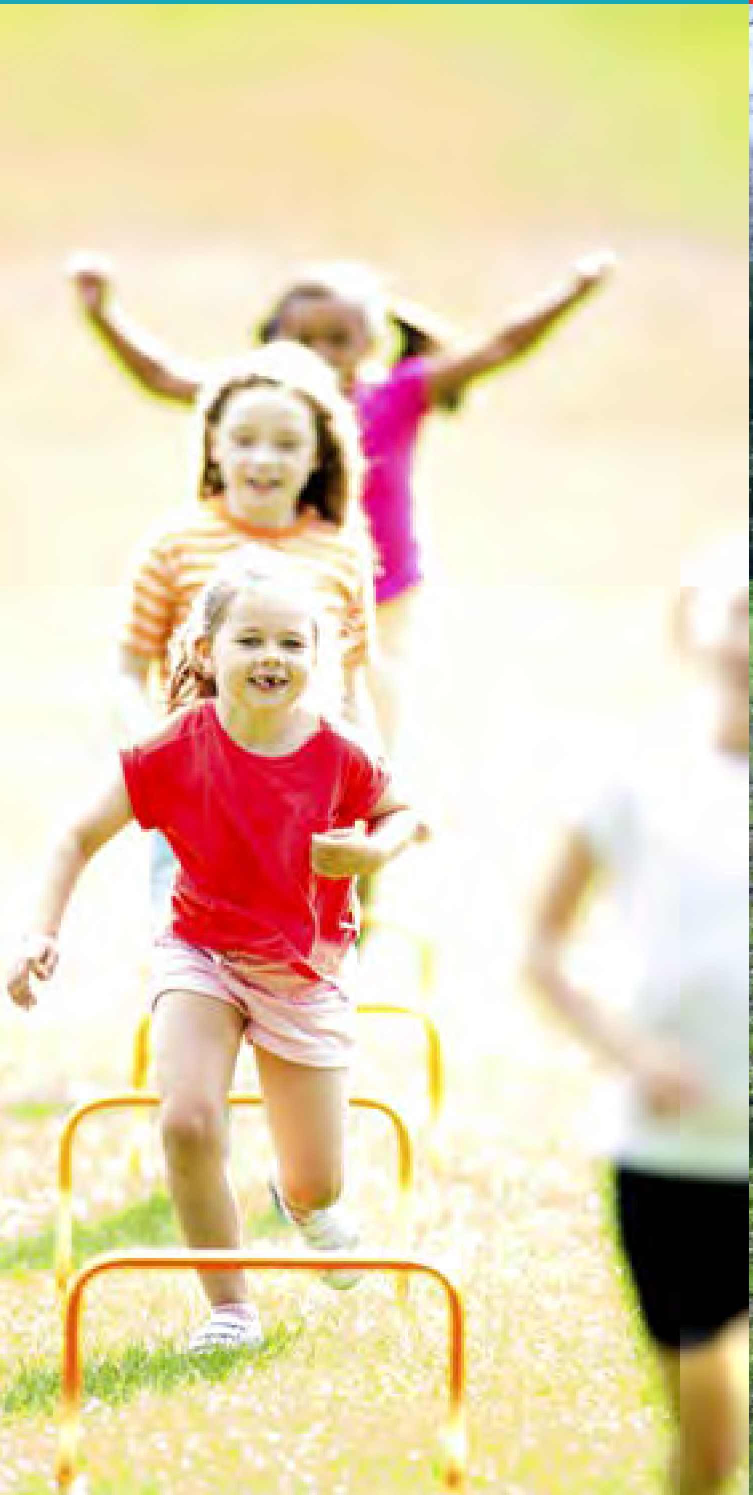

SCHOOL SPORT

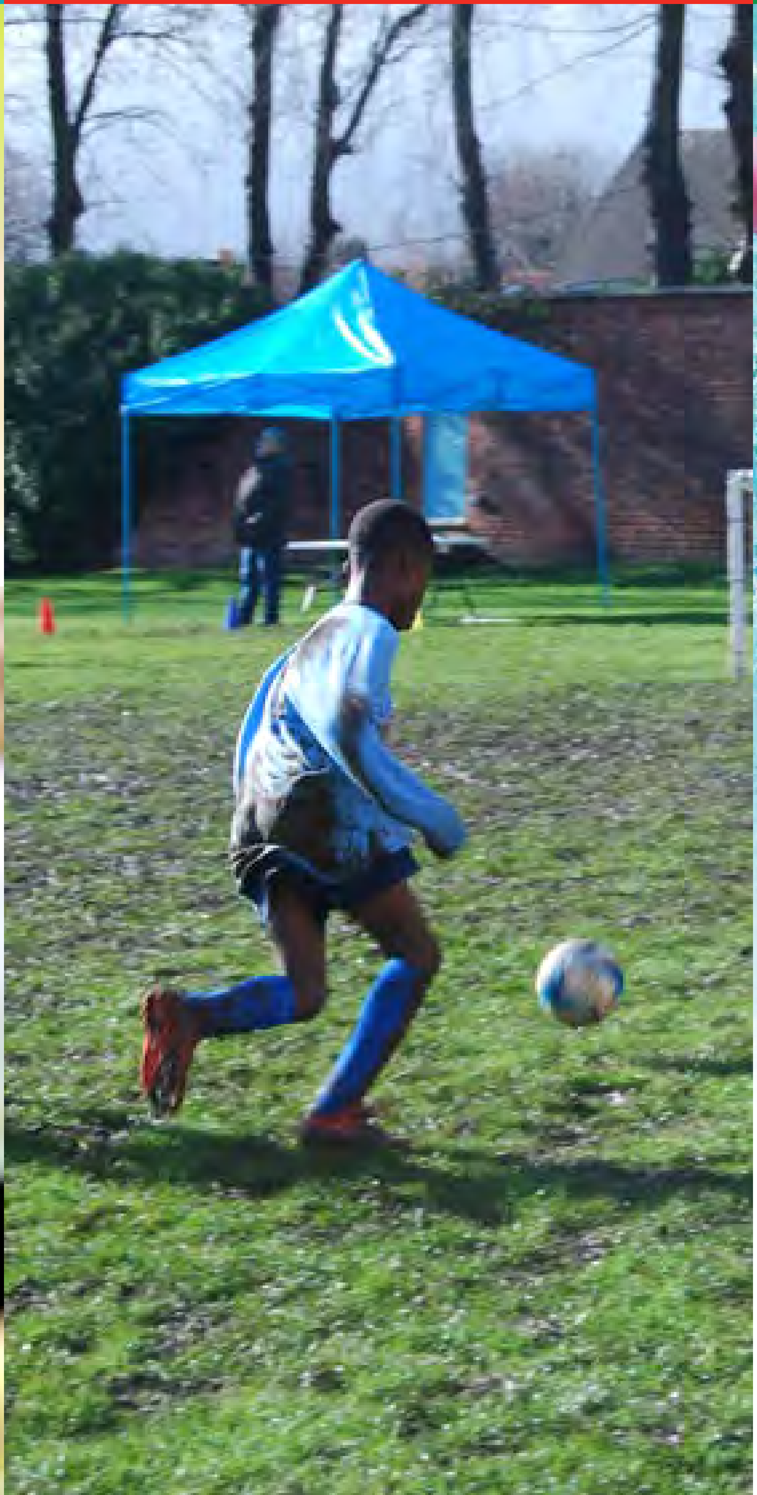

PHYSICAL ACTIVITY

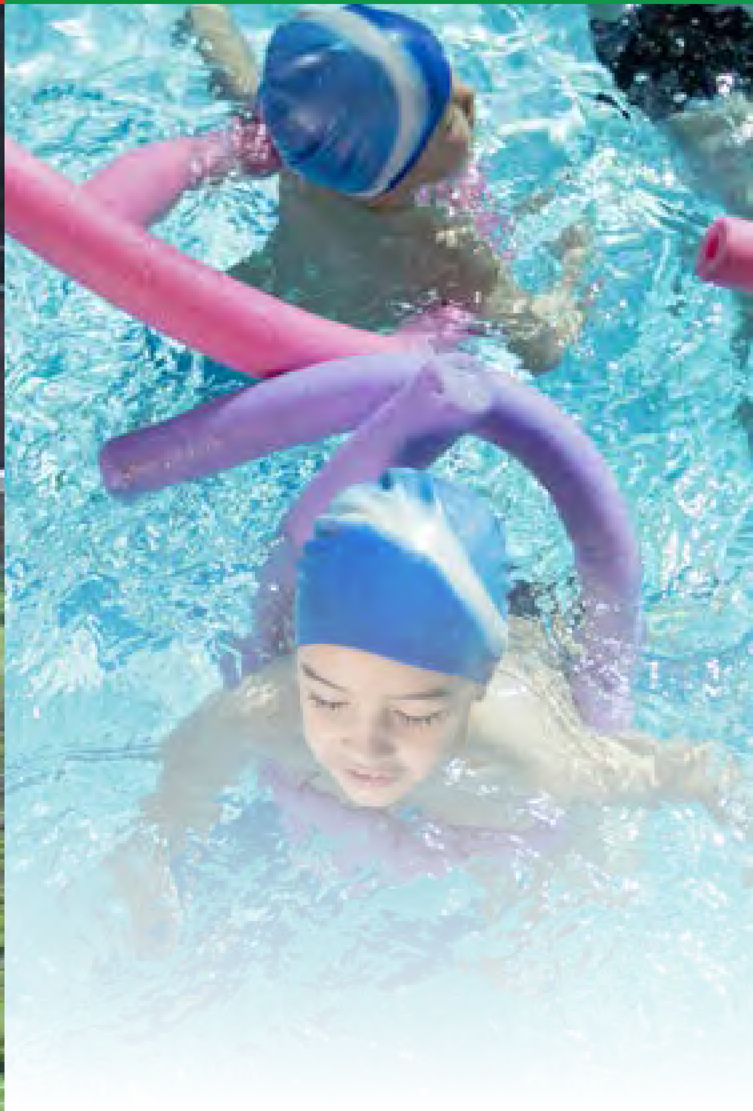

PESSPA SHEFFIELD ALLIANCE

Physical Education School Sport Physical Activity

TOOLKIT

PESSPA Toolkit
Contents

**Section 1 – Introduction**	
Purpose	3
Context	4 - 7
Vision	8
Priorities	8
**Section 2 – Pledge Statements**	9
**Section 3 – Pledge Guidance**	
Physical Education	10 - 12
School Sport	13 - 15
Physical Activity	16 - 18
**Section 4 – Audit Tool**	
Statement Descriptors	19 - 22
Audit Guidance	23 - 24
**Section 5 – Research & Impact Evaluation**	24
**Section 6 – Project Development**	25
**Section 7 – Reference List**	26 - 28

This toolkit has been developed by the Sheffield Physical Education, School Sport and Physical Activity Strategic Alliance.

This partnership includes: Learn Sheffield, Move More, the School Sport Partnerships of the city (Arches SSP, Forge SSP, Links SSP), Points Learning Network, Sheffield City Council (PESOL, Public Health and Countryside & Parks), Sheffield Hallam University, Primary PE Alliance (including school leaders from each primary locality), Sheffield Federation for School Sport, Sheffield City Trust (Sheffield International Venues), Thornbridge Outdoors, School Games Organisers, Youth Sport Trust & Yorkshire Sport Foundation.

Section 1 – Introduction

Purpose

This strategic toolkit sets out our vision for Physical Education, School Sport and Physical Activity within Sheffield. It has been developed by a strategic alliance of groups and organisations who are passionate about the value and importance of Physical Education, School Sport and Physical Activity.

We believe that:

- high quality PE, School Sport and Physical Activity are essential in preparing children and young people to live healthy lives.
- consistent access to educational establishments who are characterised by the embedded and exemplary characteristics in this toolkit will make a significant contribution to the long term health outcomes of our children and young people.
- supporting pupils to be more active in PE, Schools Sport and Physical Activity will improve our children's mental health, physical health and improve their academic attainment and achievement.

This toolkit has been designed to be used by Sheffield schools and builds on the PE Pledge which was developed and introduced in 2016. The pledge statements have been revised and now include more detailed descriptors, which provide an audit for schools to use in order to evaluate their provision and plan strategically for improvement. The toolkit also provides guidance on each aspect so that our collective understanding of the evidence base for best practice is enhanced.

The strategic alliance will continue to develop the PESSPA Toolkit using a research programme and feedback from schools. This will enable the content to support and challenge schools to improve the impact of their provision on the life chances of Sheffield's children and young people.

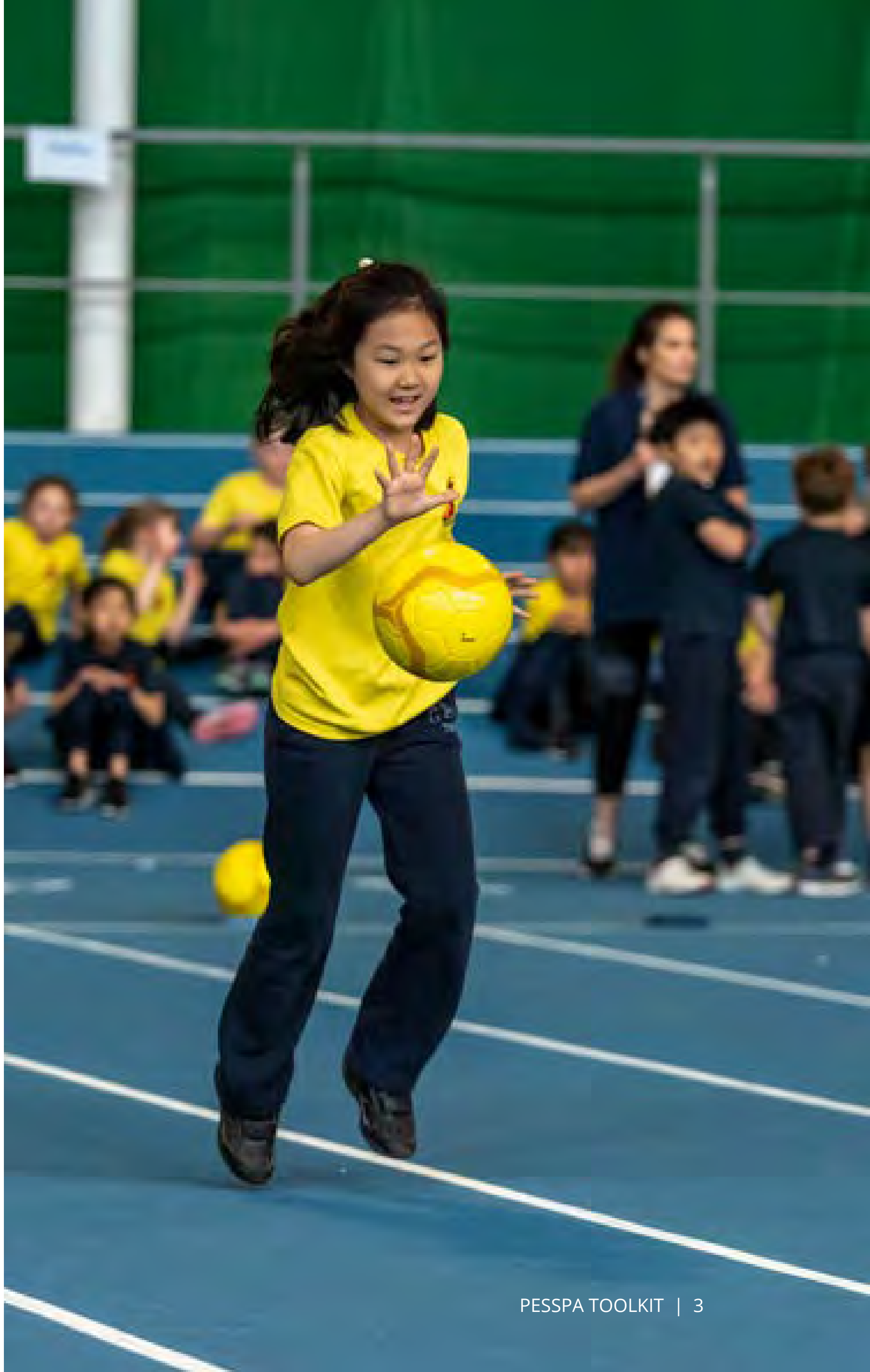

Section 1 – Introduction

Context – Physical Activity Impact

There is a strong evidence base *(Chalkley et al, 2015)* which tells us that active children and young people are more likely to have better educational outcomes including;

- improved academic achievement.
- improved cognitive functioning.
- improved attention & concentration.
- suffering less from anxiety & stress.
- improved confidence.
- improved social interactions / peer acceptance.
- improved resilience.
- improved sleep.
- improved physical fitness.
- improved motor development.
- improved classroom behaviour.

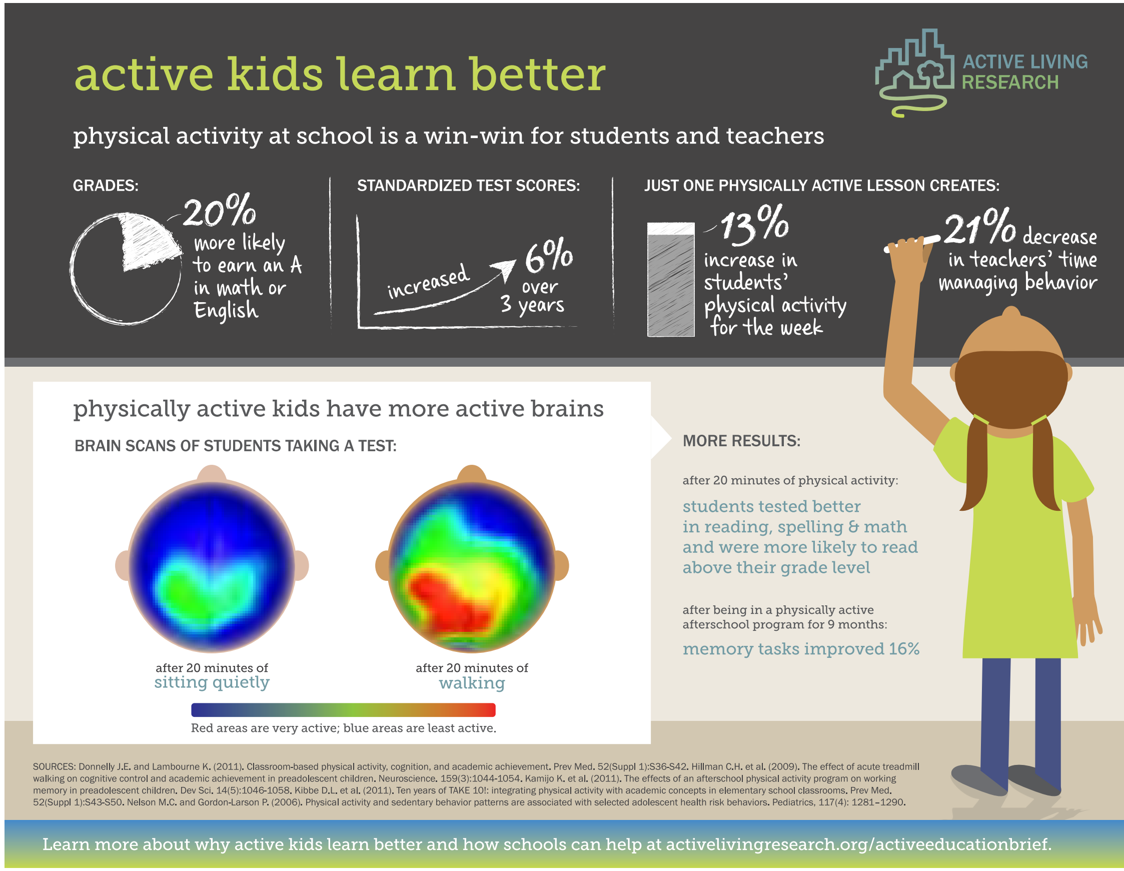

Source: Active Living Research, UC San Diego:
http://activelivingresearch.org/activeeducationinfographic

Yet, only around 20% of our children and young people meet daily physical activity recommendations (Sport England, 2018). This toolkit highlights various ways schools and staff can support our children and young people to meet daily physical activity guidelines so that they can all experience the positive benefits of being more active, improving their academic and social outcomes for life.

Section 1 – Introduction

Context – Obesity

There are significant differences between health outcomes across our city, so any strategic approach must consider how these gaps can be reduced. We recognise that the challenge of improving our children and young people's lifestyle choices is considerable and requires a step-change in our performance and mind-set.

Obesity is a key concern nationally as well as in Sheffield, with obesity found to be generally on the rise. There is also a marked difference in obesity between the most and least deprived areas of the city. This, among other health indicators has contributed to a life expectancy difference of 10 years between the most and least deprived areas of Sheffield (A Matter of Life and Healthy Life, 2016).

Obesity has risen from 15% in 1993 to 26% in 2016, with a similar story in the category 'overweight or obese', from 53% in 1991 to 61% in 2016 (House of Commons, Obesity Statistics, 2018).

One in ten Children are obese by age 5, rising to one in five being obese by the age of 11.

Sheffield year 6 obesity 2017/18 data

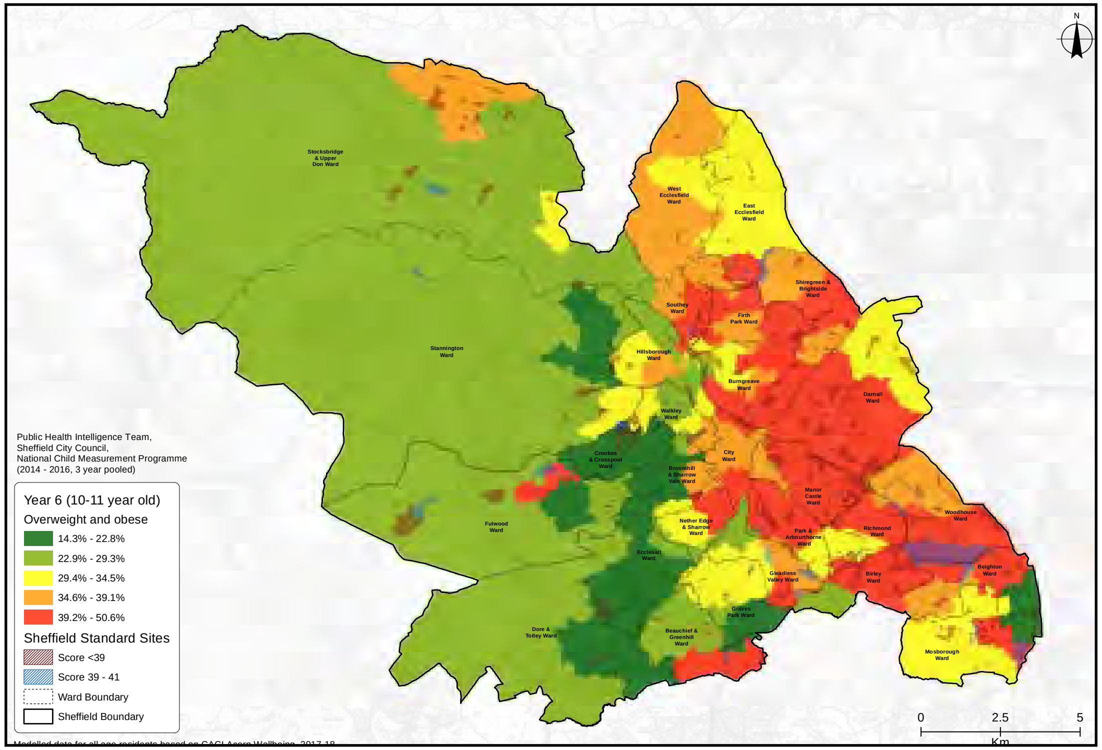

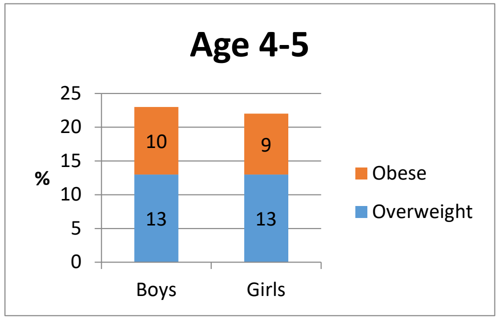

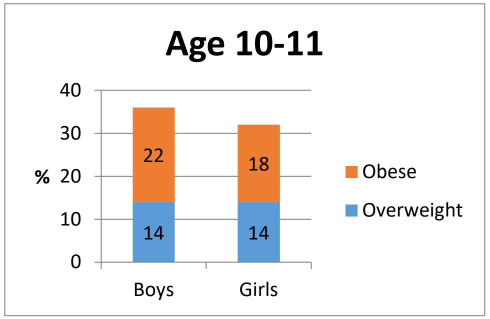

Section 1 – Introduction

Context – Physical Activity

In addition to the obesity data, Sport England's Active Lives Survey suggests that 82.5% of children and young people do not achieve Chief Medical Officer (CMO) guidelines of 60 minutes Physical Activity (PA) everyday. A breakdown analysis of that 82.5% reveal that:

- 25.7% of children are active for 60+ minutes a day, but not everday.
- 23.9% of children are active for an average of between 30 and 59 minutes a day.
- worryingly, 32.9% of children are active for less an average of 30 minutes a day.

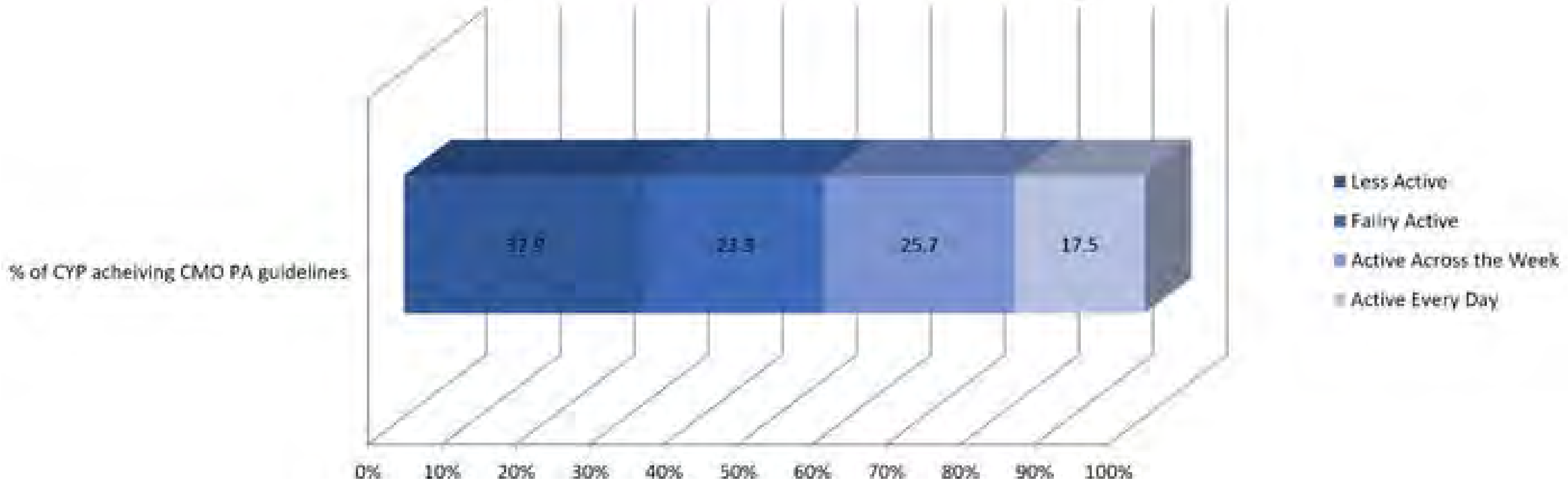

We believe that our city is uniquely placed to support children and young people to be active as part of everyday life, with the natural resources we have in Sheffield, including the parks, the outdoor city initiative, as well as the network of support schools can access through key stakeholders involved in PA promotion in Sheffield such as School Sport Partnerships / Networks, Thornbridge Outdoors.

Section 1 – Introduction

Context – Experience is Key

We know that pupils' experiences of physical education, school sport and physical activity have a significant impact on their mind-set and behaviours, both now and in the future. There is good evidence that one's (in)activity tracks into adulthood, thus it is crucial to support children and young people to be given the opportunity to make active choices built into their everyday lives at school.

The diagram below highlights various opportunities to support a child to be active throughout the school day.

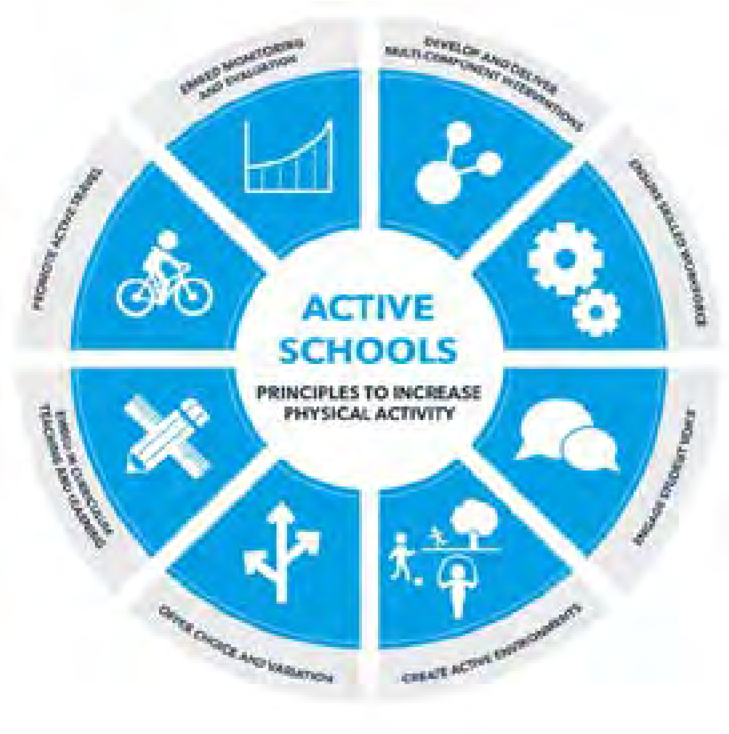

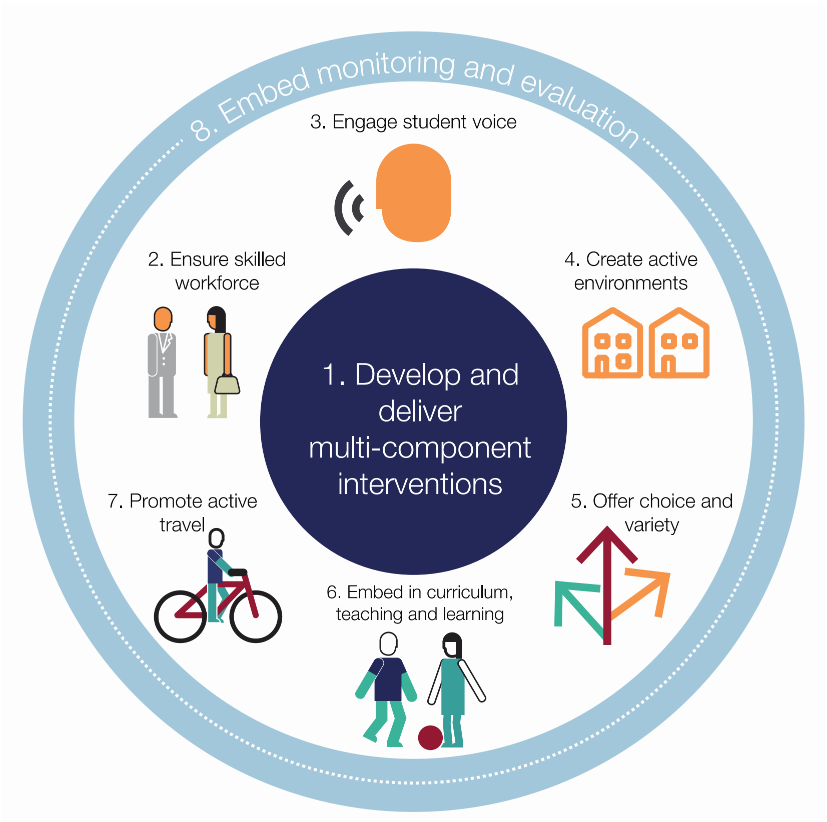

Creating enjoyable and sustainable mechanisms that your school can support pupils to be active everyday is crucial to develop children who are ready for life. The diagram above is from Public Health England's 2019 report on best practice to increase children's physical activity at school. It demonstrates eight key methods of how to engage more pupils in more physical activity, more often.

Section 1 – Introduction
Sheffield Vision

Sheffield has long valued and believed that high quality PE, School Sport and Physical Activity play an important role in preventing ill health, improving the quality of life and improving educational outcomes for children and young people within the city. This toolkit seeks to continue to raise the profile of PE, School Sport and Physical Activity in the city and to support and challenge schools and other partners to maximise the benefits which high quality provision can deliver.

In doing this we are focussed on encouraging regular sustainable participation to improve health and wellbeing; to play an important preventative role by improving the health of individuals to reduce the need for reactive and acute health services. Beyond health improvement, Sheffield strives to be a city that engages children and young people to be more active and successful citizens through participation in sport and physical activity, generating benefits through increased social cohesion, happiness of individuals and improved economic benefits.

This strategy seeks to engage every young person in Sheffield in physical activity inside and outside of school, empowering them and their families to make educated decisions about a lifelong, healthy and active lifestyle and providing them with the opportunities to take part in high quality PE, school sport and physical activity that raises their aspirations.

It will support and enable children and young people to develop the 'five ways to welling' behaviours that will support their educational ad employment outcomes, a successful transition to adulthood, and lifelong learning.

Key Outcomes & Strategic Priorities

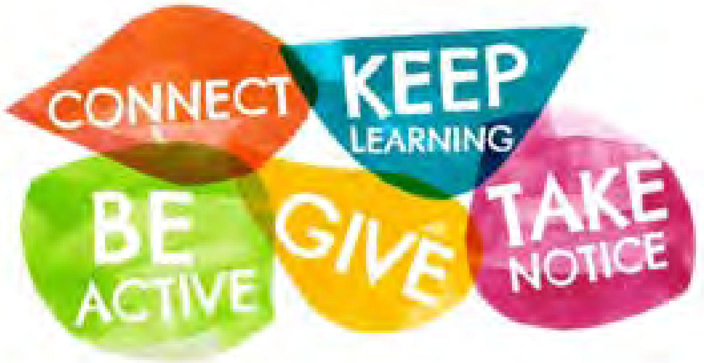

Every Sheffield pupil will benefit from:

- high quality Physical Education which develops physical literacy.
- improved opportunities and pathways to participate in school sport.
- increasing levels of physical activity throughout the school day.
- improved physical, social and emotional wellbeing.
- attending a school with a culture that recognises the value of PESSPA.
- being part of a city which gathers and shares data to evaluate impact which supports improvement planning.

Section 2 – PESSPA Pledge

Physical Education	School Sport	Physical Activity
1. Physical education, school sport and physical activity are an integral part of our school improvement culture.		
2. Our physical education, school sport and physical activity offer is inclusive and engages all pupils including the least active.		
7. Our school is committed to continually developing confidence and competence of teachers and wider school staff to enable them to deliver high quality physical education, school sport and physical activity.		
8. Our children and young people have the opportunity to learn to lead through physical education, school sport and physical activity.		
10. Our school evaluates the indicators of health and well-being for our children and uses this to inform the development of health and wellbeing provision for our school community.		
3. Our school provides two hours of high quality timetabled Physical Education per pupil per week led by a qualified teacher within the curriculum with appropriate support.	9. All children in our school participate in competition.	11. Our school has a targeted approach to improving the level of physical activity experienced by all pupils.
4. Our school works with partners to ensure all children meet the swimming National Curriculum standard before they leave primary school.		12. All pupils have the opportunity to be physically active daily within the broader curriculum, including a variety of light, moderate & vigorously intensities.
5. Physical Education is led by a subject coordinator who is a qualified teacher and supported to engage with external partners to develop high quality physical education, school sport and to promote physical activity.	14. Our school provides a range of sporting opportunities as an extension of the curriculum.	13. Our school supports pupils to achieve at least 30 minutes daily physical activity outside of the school day.
6. Our children receive Physical Education lessons which are quality assured in line with the monitoring of other curriculum areas.		

Section 3 – Pledge Guidance

Physical Education

**Overview**	High quality PE is the planned, progressive, inclusive learning experience that forms part of the curriculum in early years, primary and secondary education. In this respect, PE acts as the foundation for a lifelong engagement in physical activity and sport. The learning experience offered to children and young people through physical education lessons should be developmentally appropriate to help them acquire the psycho-motor skills, cognitive understanding, and social and emotional skills they need to lead a physically active life.<br><br>(Source: Association for Physical Education (AfPE) Health Position Paper, 2008)<br><br>Physical education combines physical competence with values-based learning and communication. When PE is taught well it provides children with a range of opportunities to develop physical confidence and competence in an environment conducive for social and emotional development.<br><br>There is a need for ongoing professional development to develop teacher confidence and competence. It is well documented that many teachers have limited ITE PE input and as a result often feel ill-equipped to teach educationally sound and safe PE lessons.
**Key Facts**	PE is not the same as Sport. This is an important distinction to make to ensure PE lessons are inclusive and meet the needs of all learners.<br><br>Teaching of PE should focus on the development of physical literacy. Physically literate children demonstrate confidence in their movement capabilities. They demonstrate agility, coordination, balance and control, and can respond to the demands of a changing environment. They work well with others and enjoy discovering new activities.<br><br>High quality PE will foster an appreciation of the intrinsic value of physical education, as well as its contribution to health and well-being. It will develop an appreciation of physical activity and will lay the foundations and values necessary for lifelong physical activity.<br><br>Quality PE provides children with a myriad of opportunities to develop social and cooperative skills and can provide them with experiences that foster self-esteem and build resilience.<br><br>Regular participation in quality physical education and other forms of physical activity can improve a child's attention span, enhance their cognitive control and speed up their cognitive processing.

	The concept of physical education is often misunderstood and as a result teaching can vary and benefits can be limited. Frequently schools judge a 'busy, happy, good' PE lesson a success irrespective of whether or not the children are learning. This of course is not just limited to learning and teaching within PE. Primary school teachers, therefore, face the ultimate paradox of teaching which is the need to manage children by keeping them controlled and quiet versus the need to provide a stimulating environment conducive to learning.
**Resources**	Department for Education (2013). National Curriculum for England. https://www.gov.uk/government/collections/national-curriculum<br><br>Association for PE (AfPE) http://www.afpe.org.uk/<br><br>Sheffield City Council, PE Swimming and Outdoor Learning Team https://www.sheffield.gov.uk/home/schools-childcare/pe-swimming-outdoor-learning.html<br><br>Youth Sport Trust https://www.youthsporttrust.org/<br><br>Cycle Experience andrew.crossley@cyclenorth.org<br><br>Links School Sport Partnership http://links-ssp.com/<br><br>Arches School Sport Partnership http://www.thearches.org.uk/<br><br>Forge School Sport Partnership https://hgcsc.co.uk/our-school/forge-school-sport-partnership<br><br>Points Learning Network http://pointslearningnetwork.com<br><br>Yorkshire Sport Foundation & Centres of Excellence https://yorkshire.sportsuite.co.uk/events/2017/01/centre-of-excellence--sports-premium-funding-allocation-and-evidencing<br><br>Thornbridge Outdoors https://www.thornbridgeoutdoors.co.uk/

**Best Practice**	Create a culture that is positive about PE and is embraced by the whole school. A culture where PE is considered important, is seen as a method to enrich the school, improve pupil learning outcomes, and aid school improvement.<br><br>Ensure PE lessons are appropriately planned, assessed and resourced and led by a qualified teacher. Lessons should be progressive and children should be aware of learning outcomes.<br><br>Quality PE focuses on areas of activity rather than the teaching of games. Children have opportunities to take part in a range of competitive, creative and challenge activities and have opportunities to be part of a team.<br><br>Quality PE encourages children to think creatively and make decisions for themselves and encourages children to reflect on their learning, fostering a desire to improve.<br><br>Quality PE is focused on the development of physical literacy and considers a range of movement opportunities rather than sport-driven activity.<br><br>The OFSTED Beyond 2012 report (2013) stated that a quarter of PE lessons were not active enough and did little to challenge children with low fitness levels or suit the needs of obese children. Children should be provided with lessons that develop specific skills while engaging with sustained physical activity.<br><br>Teachers, parents and carers are brilliant role models upon which children can model themselves. Ensure teachers model positive attitudes towards PE i.e. PE is seen as a priority and teachers dress appropriately for lessons.<br><br>PE should be enjoyable, challenging and rewarding, and generate enthusiasm for physical activity and sport.
**Local Offers and Providers**	School Sport Partnerships / Networks<br><br>Sheffield Swimming Service / Shoals Swim School<br><br>Thornbridge Outdoors

Section 3 – Pledge Guidance

School Sport

**Overview**	'School Sport is structured learning that takes place beyond the curriculum (i.e. in the extended curriculum) within school settings; this is sometimes referred to as out-of-school-hours learning. Again, the context for the learning is physical activity. The 'school sport' programme has the potential to develop and broaden the foundation learning that takes place in physical education. It also forms a vital link with 'community sport and activity' (AfPE).<br><br>School sport is an opportunity for pupils to engage with and participate within a range of sports, through a range of roles and within a range of settings. Schools should be in a position to offer a breadth of opportunity for learning and participating beyond the curriculum through their school sport offer for all pupils.
**Key Facts**	There are a large range of benefits for schools to having a robust and engaging school sport offer for pupils which are wider than raising the profile and participation in the sport itself. Pupils are able to work together with peers, improve cognitive function and communication skills, develop their resilience and increase confidence and self esteem.<br><br>School sport can help raise the profile of the school within the local community and can be used by the school as a tool for tackling specific issues across the academic year. Sport can be used as a method of allowing pupils to represent their school and feel part of the school community.<br><br>Sporting opportunities can be provided as informal activity at breaks and lunchtimes throughout the school day as well as more structured clubs being in place to target specific sports or activities. Dependant on space schools are able to offer a wide range of different sports throughout the typical school day and can offer opportunities for pupils to try sports that they would never have the chance to undertake outside of the school environment. These may be delivered by school staff or could be an opportunity for the school to engage with external partners and specific qualified deliverers to enhance their pupil's opportunities.

	Sports Clubs are a crucial tool in terms of delivering sporting opportunities to pupils across the school. These clubs should be seen as an opportunity for pupils to further develop their skills. Schools should analyse the access that their pupils have to these on a regular basis to ensure that targeted opportunities are able to be developed where necessary.<br><br>Opportunities to lead activities and other varied roles can be provided through sport within a school setting. Sport can provide opportunities for pupils to experience delivering coaching and officiating for example as opposed to engaging solely as a participant. Leadership can take place in other forms across school in terms of wider roles (sports council or sports journalist for example) and this can be used to target and support more disengaged pupils or pupils who are excelling within a particular activity/ sport.<br><br>Competition can be introduced using sport as a tool across school and is it encouraged for all schools to embed this process within their school for every child. This can take place in terms of personal challenges and improving on scores previously (competition against self) or can be implemented using intra school competition against others, either personally or part of a team.<br><br>The above will allow for schools to then further engage in competitions or multi school events known as Inter School Competitions. These may be more local events (such as a friendly match against the next school for example) or may be much larger scale sporting events where schools can take part. In addition to competitive opportunities, schools may also take part in more skill based festivals where pupils are able to experience a new sport or develop their ability levels. It is important for schools to assess the needs of their pupils and ensure that the appropriate opportunities are provided.<br><br>It is essential that schools are able to work with external partners to ensure that sustainable opportunities are provided for pupils to participate in specific sports where possible. Schools should work with local community sports clubs and other local opportunities where possible and highlight these to pupils to ensure that children and families are aware of these.
**Resources**	Association for PE (AfPE)<br>Youth Sport Trust<br>School Games<br>Sports Leaders UK

**Best Practice**	School sport should be a factor in the development of the school and used as a catalyst to support whole school improvement changes wherever possible. There should be a wide range of opportunities for all pupils to engage in sport across the academic year in a way which suits their individual needs and abilities.<br><br>A range of formal and informal opportunities should be provided daily for pupils and targeted support should be offered to engage specific pupils where appropriate. Pupil voice should be used throughout the academic year to analyse the interests of pupils and sports then offered to pupils where possible, either within school or outside of the school environment. Pupils should be encouraged to try alternative activities and put themselves in environments that they may not be used to, to provide a sporting experience for them.<br><br>A large majority of pupils will be offered the opportunity to lead their peers within a sporting activity and in a varied number of roles.<br><br>Schools should engage in a wide range of competition within school (intra school) and also against other schools (inter school) to ensure that all pupils are able to compete at a level that suits them. It is important that a range of different sports are offered and that the school work closely with external partners to evaluate what they can engage with and the suitability for pupils to ensure a positive experience. The principles of competition should involve safe practice, maximum participation, inclusiveness, learning for all, enjoyment and that all pupils experience success.
**Local Offers and Providers**	School Sport Partnerships / Networks<br><br>Local School Games Organisers<br><br>Local Community Sports Clubs<br><br>Professional Clubs Community Bodies

Section 3 – Pledge Guidance

Physical Activity

**Overview**	Physical Activity (PA) is defined as bodily movement produced by skeletal muscles that require energy expenditure *(WHO, 2010)*. PA is a well-established preventative for non-communicable diseases (NCD) such as heart disease, stroke, diabetes, and cancer *(WHO, 2010)*, a preventative for NCD risk factors such as obesity and hypertension *(Schuch et al, 2016)*, and has significant associations with improved mental health, delay in the onset of dementia, and improved wellbeing and quality of life *(Das & Horton 2012; Livingston et al, 2017; Mammen, 2013; WHO, 2018)*.<br><br>PA is a central aspect of both ones mental and physical health and makes up part of a healthy lifestyle *(WHO(a), 2018)*. Unfortunately however, only 21% of children and young people are achieving sufficient daily PA *(Townsend et al, 2015)*, which is likely to have significant detrimental consequences on children and young people's physical and mental health.<br><br>There is a need for intervention to help improve the health and wellbeing of our children and young people. Schools are a fantastic time in which to intervene as pupils are a captive audience for around six hours a day. Research supports the positive impact an intervention can have in such places (e.g. schools, workplaces) *(GAPA, 2011)*.
**Key Facts**	PA, PE, and Sport are not the same thing, rather three different ways in which to be active, whilst each being critical in developing children to be ready for life. This is an important distinction to make as the lines between the three are often blurred.<br><br>• PE is defined as the 'planned, progressive learning that takes place in the school curriculum timetabled time and involves learning to move (i.e. becoming more physically competent) and moving to learn (i.e. learning skills such as cooperation through movement)'.<br>• Sport, is defined as 'an activity involving physical exertion and skill in which an individual or team competes against another or others for entertainment' *(Andriyani, 2014)*.<br>• PA however, as previously defined, is merely the creation of bodily movement and is not dependent on competition *(Andriyani, 2014)* or specific learning outcomes, rather PA can be used to enhance learning outcomes.<br><br>Children and young people should be active for at least 60 minutes per day at a moderate to vigorous intensity, as stated by the Chief Medical Officer (CMO) *(Start Active, Stay Active, 2018)*. However, only 21% of children and

	young people meet this guideline *(Townsend et al, 2015)*. This high prevalence of physical inactivity is likely to be a significant contributing factor to an astonishing 33% of children leaving primary school overweight or obese *(Making Obesity Everybody's Business, 2017)*, with physical activity being one of the active ingredients to helping manage one's weight. Child fitness levels are currently unknown on a national scale.<br><br>A person's cardio respiratory fitness, which is strongly linked to one's physical activity, is the strongest predictor for a healthy and long life, proving significantly more important that obesity *(Blaire, 2009)*. Physical inactivity has been identified as the fourth leading risk factor for global mortality (6% of deaths globally) *(WHO(b) 2018)*.<br><br>PA has a strong relationship with mental health, meaning sufficiently increasing a child's physical activity could significantly reduce the likelihood of a child suffering from mental health issues. Physically active people of all ages, socioeconomic groups, and ethnicities are happier, healthier and more productive compared to sedentary peers *(The Move More plan, 2015)*.<br><br>More active, fitter children have been found to have;<br>• Improved academic performance.<br>• Improved concentration and focus on task.<br>• Improved mental and physical wellbeing.<br>• Improved social experiences.<br>• Improved skill/motor development.<br>• Improved behaviour.<br>*(Chalkley et al, 2015)*
**Resources**	For up to date resources or explanations please see the following links, depending on your wants and needs:<br>• Learn Sheffield: How Physical Activity will drive school improvement.<br>• Move More: Sheffield's strategic Physical Activity Plan for PA, PE, and School Sport.<br>• SSP (School Sport Partnership and Learning Network): Provider knowledge and providers of PA, PE, and School Games resources for Sheffield.<br>• PESOL (PE, Swimming and Outdoor Learning): Knowledge of available resources and avenues for PE, swimming and outdoor learning experiences.<br>• The Outdoor City & the Sheffield Rotherham Wildlife Trust are great avenues for being pointed in the right direction to explore the great outdoors.

**Best Practice**	Create a culture within your school where PA is considered important, seen as a method to enrich the school, improve pupil learning outcomes, aid in school improvement, and improve child readiness for life. Developing good PA habits in a child increases the likelihood for improved physical and mental health outcomes, enriching a child's readiness for life. In order to do this, the management team, teaching staff and supporting staff all need to deliver a clear message, encouraging pupils that PA is the norm, so every pupil feels safe, confident, and comfortable in which to be active, and to participate in PA opportunities throughout the school day.<br><br>Think of the physical environment in which pupils operate in within a school day; is this optimised to allow pupils to be active? Providing resources or provision at break times can really help increase pupil's daily PA levels.<br><br>PA is not just for the PE lead/department. PA should be on every member of staff's agenda. This will help create a positive activity culture within your school, helping pupils perception of PA to be important in all elements of life, and that activity is not only PE, rather PE is one element of PA. Teachers and parents are brilliant role models for children to model themselves on. Engaging teachers and parents in regular PA will have a positive impact on pupil's perception on PA. Active Commuting, teachers joining in during active break times, and active learning are great examples of this.<br><br>Allow the pupils to help steer what PA initiatives there are in the school. Co-production has proven to be a powerful tool to increase motivation and participation. The pupils will also likely come up with some new and exciting ideas to get active both in the classroom and break times.<br><br>Clearly define what PA is as children and young people will all have different levels of understanding. PA is defined as any bodily movement produced by skeletal muscles that requires energy expenditure, encompassing any movement.<br><br>The key is regular PA everyday, with a mixture of light, moderate, and vigorous PA. We see the greatest return on mental and physical health from moderate to vigorous PA and so incorporating these intensity's into each school day is important. Reducing sedentary time or breaking sedentary time up with light PA is also of great benefit. Examples of getting pupils active in the classroom include active brain breaks and physical activity learning.
**Local Offers and Providers**	There are many ways to embed physical activity into everyday school life, including active lessons, active breaks, active travel etc.<br><br>For more information, get in contact with your School Sport Partnerships / Networks

Section 4 – Audit Tool

Physical Education, School Sport & Physical Activity – Pledge Statements & Descriptors

The descriptors below are designed to exemplify each of the pledge statements. In each case the statement itself forms the embedded descriptor, with the emerging and establishing descriptors describing the steps towards achieving the pledge and the exemplary statement providing an example of the practice in that area that would be notable and should be shared.

Pledge Statement	Emerging (practice at an early stage)	Establishing (practice in place)	Embedded (practice robustly in place)	Exemplary (practice that should be shared)
1. Physical education, school sport and physical activity are an integral part of our school improvement culture.	Our school has an improvement plan for physical education, school sport and physical activity.	Physical education, school sport and physical activity are visual within school for all stakeholders.	Physical education, school sport and physical activity are an integral part of our school improvement culture.	Physical education, school sport and physical activity are fundamental to our whole school culture and embedded in all aspects of school life.
2. Our physical education, school sport and physical activity offer is inclusive and engages all pupils including the least active.	Our school ensures that all pupils are engaged and included. Provision is starting to meet differing needs and opportunities are offered to all pupils.	Systems of monitoring all pupils (including most able, least active...) acted upon by staff in lessons, physical activity and school sport by ensuring appropriate differentiation. Personal challenge is increasingly a feature of provision.	Our physical education, school sport and physical activity offer is inclusive and engages all pupils including the least active.	Identified groups of pupils have a voice and can influence physical education school sport and physical activity. All pupils are aware of what they must do to improve and refer often to their own personal physical challenges.
3. Our school provides two hours of high quality timetabled Physical Education per pupil per week led by a qualified teacher within the curriculum with appropriate support.	Our school provides all pupils with two hours of Physical Education per week timetabled within curriculum time.	Our school monitors provision to ensure that all pupils access two hours of increasingly high quality Physical Education per week led by a qualified teacher within the curriculum with appropriate support.	Our school provides two hours of high quality timetabled Physical Education per pupil per week led by a qualified teacher within the curriculum with appropriate support.	Our school consistently provides all pupils with two hours of high quality timetabled Physical Education regardless of other events, activities, priorities in any given week taught by a qualified teacher

Pledge Statement	Emerging (practice at an early stage)	Establishing (practice in place)	Embedded (practice robustly in place)	Exemplary (practice that should be shared)
4. Our school works with partners to ensure all children meet the swimming National Curriculum standard before they leave primary school.	All pupils have the opportunity to learn to swim before they leave primary school. Swimming results are published on the school website.	The School works alongside swimming providers to ensure that lessons are meeting the needs of all pupils. Children and parents/carers are given access to information prior to swimming lessons which will prepare them to learn.	Our school works with partners to ensure all children meet the swimming National Curriculum standard before they leave primary school.	Continual evaluation of the school swimming provision allows all pupils make progress. Additional opportunities may be provided to enable all pupils to achieve the standard. School engages with partners to provide pathways to participation outside school and enter competitive opportunities.
5. Physical Education is led by a subject coordinator who is a qualified teacher and supported to engage with external partners to develop high quality physical education, school sport and to promote physical activity.	Physical Education is led by a subject co-ordinator who reports to school leaders and governors about the quality of provision.	Physical Education is led by a subject co-ordinator who is actively supported by school leaders and given some dedicated time to quality assure and support the improvement of provision.	Physical Education is led by a subject co-ordinator who is a qualified teacher and supported to engage with external partners to develop high quality physical education, school sport and to promote physical activity.	The Physical Education co-ordinator is a school leader who drives high quality physical education, school sport and promotes physical activity. The culture of the school supports all aspects of provision.
6. Our children receive Physical Education lessons which are quality assured in line with the monitoring of other curriculum areas.	Monitoring of Physical Education lessons is taking place to develop an understanding of the quality of Physical Education teaching across the school.	Systematic monitoring of Physical Education lessons, often involving the co-ordinator and school leaders, ensures that a robust understanding of the quality of teaching across the school exists.	Our children receive Physical Education lessons which are quality assured in line with the monitoring of other curriculum areas.	Physical Education is routinely monitored with effective systems that support consistently high quality teaching of Physical Education.
7. Our school is committed to continually developing confidence and competence of teachers and wider school staff to enable them to deliver high quality physical education, school sport and physical activity.	Staff who teach Physical Education regularly complete an audit of their development needs. The school CPD offer is developed to meet identified needs.	All staff are actively encouraged to attend targeted and strategically identified CPD which addresses their identified development needs. Some CPD is provided to develop wider school staff.	Our school is committed to continually developing confidence and competence of teachers and wider school staff to enable them to deliver high quality physical education, school sport and physical activity.	CPD is regular, sustainable, monitored and accessed by all staff leading to high quality teaching and learning.

Pledge Statement	Emerging (practice at an early stage)	Establishing (practice in place)	Embedded (practice robustly in place)	Exemplary (practice that should be shared)
8. Our children and young people have the opportunity to learn to lead through physical education, school sport and physical activity.	Opportunities are in place for pupils to learn to lead within physical education, school sport and physical activity in school. This may be most developed in curriculum activities, for example leading a partner in Physical Education.	Increasing opportunities are in place for pupils to learn to lead within Physical Education lessons and across school sport and physical activity in school. Development of sports leaders includes activities at playtimes and outside the curriculum.	Our children and young people have the opportunity to learn to lead through physical education, school sport and physical activity.	Leadership is a fundamental part of all physical education, school sport and physical activity which impacts on other curriculum areas and pupil behaviours. Pupils systematically take part in a variety of leadership roles throughout their time at the school
9. All children in our school participate in competition.	A number of competitive opportunities are provided for our pupils throughout lessons and school provide opportunities for a cohort of pupils to represent the school.	Pupils are encouraged to engage in competition regularly throughout our school sport offer which includes all pupils taking part in Personal Challenge and pupils engaging in Intra School Competition. We offer a range of opportunities for pupils to compete for and represent our school	All children in our school participate in competition.	The school provides a wide range of opportunities for all pupils to participate in competition. Pupils are targeted to ensure appropriate opportunities for all. Pupils are able to express why they compete and the school fully engages with the principles of competition.
10. Our school evaluates the indicators of health and well-being for our children and uses this to inform the development of health and wellbeing provision for our school community.	Our school provides clear messages about health and wellbeing to both children and their parents/ carers. Staff and governors have an increasing understanding of key indicators and the appropriate methods that we can use to evaluate them.	Our school has a developing understanding of health and wellbeing indicators, which are increasingly shared with both children and their parents/ carers. Evaluation of health and wellbeing is beginning to inform the development of our provision.	Our school evaluates the indicators of health and well-being for our children and uses this to inform the development of health and wellbeing provision for our school community.	Our school community has a shared understanding of health and wellbeing indicators. Children are able to self-report about their health and wellbeing and opportunities are provided for this to be combined with adult observations in order to evaluate progress and identify appropriate provision.

Pledge Statement	Emerging (practice at an early stage)	Establishing (practice in place)	Embedded (practice robustly in place)	Exemplary (practice that should be shared)
11. Our school has a targeted approach to improving the level of physical activity expe-rienced by all pupils.	Our school has an increasing awareness of the importance of physical activity and the key themes in the development of opportunities for pupils to be more physically active.	Our whole school plan for physical activity includes the physical environment, social environment and the range of opportunities to be active. Monitoring is starting to lead to targeted opportunities for the least active pupils.	Our school has a targeted ap-proach to improving the level of physical activity experienced by all pupils.	Our embedded targeted approach results in active staff and pupils having the opportu-nity to devise and engage in a wide range of physical activity. Appropriate targeting ensures that all pupils are physically active.
12. All pupils have the oppor-tunity to be physically active daily within the broader cur-riculum. Including a variety of light, moderate & vigorously intensities.	Resources and tools are shared across the school to all teach-ers, increasing awareness and planning of how physical activity can be implemented into their planning of learning experienc-es.	The majority of teachers are successfully implementing aspects of physical activity into their classroom each day.<br><br>Best practice is shared across the school from those who have managed to successfully embed physical activity.	All pupils have the opportunity to be physically active daily within the broader curriculum. Including a variety of light, mod-erate & vigorously intensities.	Opportunities to be physi-cally active are frequent and varied in all classrooms across the school. Teachers regard physical activity as a teaching tool and professional dialogue supports rapid sharing of inno-vative practice.
13. Our school supports pupils to achieve at least 30 minutes daily physical activity outside of the school day.	Our school communicates sup-portively with parents and carers about the importance ensuring that children meet guidelines of 30 minutes’ activity outside the school day.	Our school has a clear strategy to support parents and carers to help their children to achieve 30 minutes’ physical activity outside the school day. Monitoring of the strategy is starting to lead to targeted support for families.	Our school supports pupils to achieve at least 30 minutes daily physical activity outside of the school day.	Our school community has a shared understanding of the im-portance of physical activity and how all children will achieve 30 minutes’ physical activity, includ-ing targeted pupils. Key outcomes reflect the priority for our school.
14. Our school provide a range of sporting opportunities as an extension of the curriculum	Our school offers a number of sports clubs and activities across the academic year which pupils are encouraged to attend.	Sports clubs and activities take place at school regularly and at-tendance at these is monitored. A wide range of sports are available for pupils to experiences which enable pupils to explore their relationship to sport.	Our school provides a range of sporting opportunities as an extension of the curriculum.	A varied extra-curricular offer is developed in partnership with pu-pils. Careful monitoring of the at-tendance of all pupils in extra-cur-ricular opportunities to ensure that appropriate opportunities are provided for all, including the least active.

Section 4 – Audit Tool (continued)

Audit Guidance

This audit tool has been designed to support schools to evaluate the quality and impact of PESSPA provision.

This tool could be used in a number of different ways and some examples of this are listed below.

- Internal desktop review - school leaders and/or governance leaders may wish to use this audit to provide an overview of Physical Education, school sport and physical activity based on existing sources of evidence and knowledge.
- Internal review trail – subject leader (potentially supported by other leaders) may wish to use this audit as a framework for a review trail within school, using some of the sources of evidence below to inform judgements against the statement descriptors and identify next steps for the school.
- Peer Review – subject or school leaders may wish to use this audit alongside colleagues from other schools. Using a common framework and similar sources of evidence will provide enhanced opportunities for professional dialogue. A programme of peer reviews fo this type across a group of schools can be used to identify strong practice as well as identify areas for improvement for individual schools or groups.
- External Review – subject or school leaders may wish to use this audit alongside external colleagues in order to benefit from their additional expertise. External colleagues who have the opportunity to work with multiple schools can provide judgements which are more 'bench-marked' and are more likely to be able to suggest improvements from their experience of other schools.

A number of different sources of evidence can be used to inform the judgements made by school leaders about the statements and descriptors. These may include:

- Pupil voice through talking to pupils or using pupil questionnaires (including attitudinal information).
- Observations from lessons observations or school walks (including collecting evidence relating to the use of physical activity as a teaching tool).
- Observations from learning walks (including displays and the school environment).
- Staff perspectives gathered through discussion or using questionnaires.
- Parent and carer voice gathered through discussion or using questionnaires.
- Available or collected data (for example relating to National Curriculum requirements, School Sport participation, staff training records or engagement with extra-curricular opportunities) and contextual data (including attendance and protected characteristics data).

- Review of (subject or school) action plans and budget tracking (for example linked to the website reporting tool evidencing the impact of primary PE and Sports Premium).
- Analysis of timetables or other records (including meeting minutes).
- School communications (including the website and other communication tools).

In completing the audit, school leaders may wish to identify and consider key review questions and having completed the audit, school leaders may wish to consider key reflection questions. The Sheffield PESSPA Alliance intend to develop case study examples of how the audit tool has been used to support colleagues in planning their own activities.

We would also ask Sheffield schools to consider and support the research programme that has been developed to review the impact of this tool and inform the future development of resources and activities within the city. You can support the research programme by:

- Sharing your completed audit with us – which will enable us to build a picture of city provision, strengths and areas of need.
- Providing feedback on the audit tool – including examples and case studies that can be shared.
- Supporting colleagues through the use of peer review and professional dialogue to enhance the feedback that can be provided.
- Volunteering to support the research programme outlined below.

Section 5 – Research Programme

It is the view of the stakeholders involved in the development of the toolkit that we need to evidence the impact of the work we do to inform future work and collaborations. In order to complete this, Move More, Sheffield Hallam University and Learn Sheffield have teamed up to research and evaluate the toolkit.

Utilising formative evaluation techniques to understanding in the process of the tool creation, process evaluation to gain an insight into how the tool is used, impact evaluation to understand the impact of the tool on school pupils and staff, and finally, research the wider themes of implementation and impact of the project will inform learning and future work.

Section 6 – Project Development

This project originated through the PE alliance group in Sheffield, who created the PE Pledge for the primary school sector. With the success of this, and with an ever-changing landscape and culture, Sheffield recognised the potential impact this tool could have with a wider scope, incorporating school sport and physical activity as well. This toolkit was developed by teachers, researchers, and significant organisations involved in PE, School Sport and Physical Activity in Sheffield. The team of people and organisations will continue to work together to develop this and other similar ideas in the future to better the physical and mental health of our children and young people in Sheffield using physical activity, leading locally and nationally as a way to address the growing physical and mental health issues in today's society.

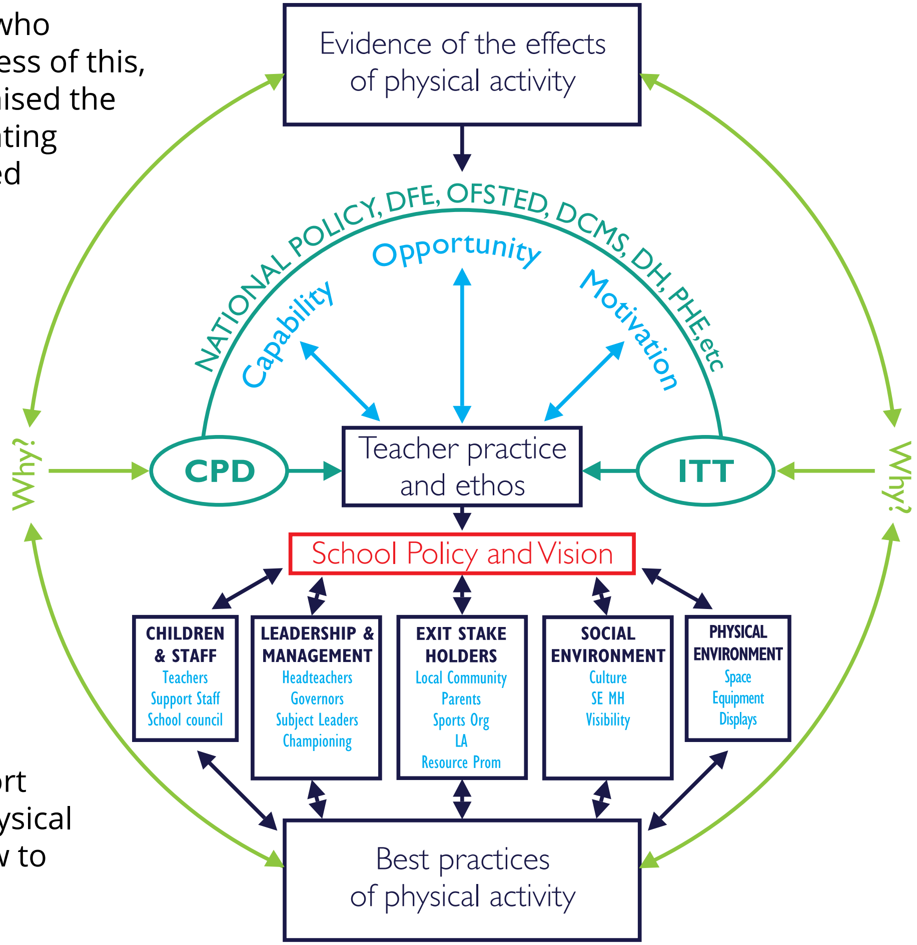

The diagram to the right is a framework that was developed through Yorkshire Sport Foundation and key partners. Sheffield has adopted a very similar working ethos and framework to support Sheffield schools to create system change in school population physical activity. The present document represents a working toolkit of how to translate this framework into action through the self-audit tool.

Section 7 – Reference List and Further Recommended Reading

- A Matter of Life and Healthy Life (2016). Director of Public Health Report 2016. Available at: https://www.sheffieldccg.nhs.uk/Downloads/About%20US/CCG%20Governing%20Body%20Papers/2016/6%20October%202016%20GBP/Item%2022k%20Director%20of%20Public%20Health%20report.pdf
- Andriyani, F. D. (2014) Physical activity guidelines for children. Jurnal Pendidikan Jasmani Indonesia, Vol.10(1),; 61-67.
- Blaire, S. N. (2009) Physical inactivity: the biggest public health problem of the 21st century. BR J Sports Med, vol.43(1).
- Chalkley, A., Milton, K., Foster, C. (2015). Change4Life Evidence Review: Rapid evidence review on the effect of physical activity participation among children aged 5 – 11 years. London: Public Health England
- Creating a culture of physical activity; The Move More Plan. A framework for increasing physical activity in Sheffield 2015-2020. Available at: (https://www.movemoresheffield.com/Media/Default/Documents/move-more-plan.pdf)
- Das, P. & Horton, R. Rethinking our approach to physical activity. Lancet. 2012;380(9838):189–190.
- Department for Education (2013). National Curriculum for England. Available at: https://www.gov.uk/government/collections/national-curriculum
- Global Advocacy for Physical Activity (GAPA) the Advocacy Council of the International Society for Physical Activity and Health (ISPAH). NCD Prevention: Investments that Work for Physical Activity. February 2011. Available at: www.globalpa.org.uk/investmentsthatwork
- Griggs, G. (2010). For sale – primary physical education. £20 per hour or nearest offer. Education 3-13. International Journal of Primary, Elementary and Early Years Education, 38 (1): 39-46.
- Griggs, G. (2016). Spending the primary physical education and sport premium: a West Midlands case study. 3-13. International Journal of Primary, Elementary and Early Years Education, 44 (5): 547-555.
- Harris, J., (2018) The Case for Physical Education becoming a Core Subject in the National Curriculum. Available at: http://www.afpe.org.uk/physical-education/wp-content/uploads/PE-Core-Subject-Paper-20-3-18.pdf
- Harris, J., & Cale, L. (2018). Promoting active lifestyles in schools. Champaign, IL: Human Kinetics.

- House of Commons Library (2019). Obesity Statistic, Briefing paper. Available at: https://researchbriefings.files.parliament.uk/documents/SN03336/SN03336.pdf
- International Council of Sport Science and Physical Education (ICSSPE) (2010). International position statement on physical education. https://m.paralympic.org/sites/default/files/document/120203172924430_2_7_2_B_Attachment_ICSSPE_International_Position_Statement_on_Physical_Education.pdf
- Livingston G, Sommerlad A, Orgeta V, Costafreda SG, Huntley J, Ames D, et al. Dementia prevention, intervention, and care. Lancet. 2017;16;390(10113):2673–2734.
- Making obesity everybody's business: A whole system approach to obesity (2017). Local Government Association, Public Health England. Available at: https://www.local.gov.uk/sites/default/files/documents/15.6%20Obesity-05.pdf
- Mammen G, Faulkner G. Physical activity and the prevention of depression: a systematic review of prospective studies. Am J Prev Med. 2013;45(5):649– 657.
- Ofsted (2013). Beyond 2012 – outstanding physical education for all. Physical education in schools 2008-12. www.ofsted.gov.uk
- Ofsted (2017). HMCI's commentary; recent primary and secondary curriculum research. Available at: https://www.gov.uk/government/people/amanda-spiel-man
- Public Health England (2014). The link between pupil health and wellbeing and attainment. A briefing for headteachers, governors and staff in education settings. PHE publications gateway number: 2014491.
- Public Health England (2019). What works in schools and colleges to increase physical activity? Available at: https://assets.publishing.service.gov.uk/government/uploads/system/uploads/attachment_data/file/821463/What_works_in_schools_and_colleges_to_increase_physical_activity.pdf
- Schuch, F., Vancampfort, D., Richards, J., Rosenbaum, S., Ward, P. B., Stubbs, B. Exercise as a treatment for depression: a meta-analysis adjusting for publication bias. J Psychiatr Res. 2016;77:42–51.
- Smith, A. (2015). Primary school physical education and sports coaches: evidence from a study of School Sport Partnerships in north-west England. Sport, Education and Society, 20 (7): 872-888.
- Sport England (2018). Sporting Future. Second Annual Report. HM Government.
- Start active, stay active: infographics on physical activity (2018). Available at: https://www.gov.uk/government/publications/start-active-stay-active-infographics-on-physical-activity

- Sport England (2018). Active Lives Children and Young Peoples Survey, Academic Year 2017/18. Available at: http://www.sportengland.org/media/13698/active-lives-children-survey-academic-year-17-18.pdf
- The Telegraph (2018). PE is a more important school subject than history, survey by YouGov finds. Helena Horton. 15th February 2018. 20th March 2018 Page 8
- Townsend N, Wickramasinghe K, Williams J, Bhatnagar P, Rayner M (2015). Physical Activity Statistics 2015. British Heart Foundation: London.
- Welsh Government (2013). Physical literacy – an all-Wales approach to increasing levels of physical activity for children and young people. Cardiff: Schools and Physical Activity Task and Finish Group.
- WHO (2010). Global recommendations on physical activity for health. Geneva: World Health Organization.
- WHO(a) (2018). Global action plan on physical activity 2018–2030: More active people for a healthier world. Geneva: World Health Organization; 2018. Licence: CC BY-NC-SA 3.0 IG.
- WHO(b) (2018). Global Strategy on Diet, Physical Activity, and Health. Available at: http://www.who.int/dietphysicalactivity/pa/en/
